# Supplementary figures and images for: Inhibition of innate immune response ameliorates Zika virus-induced neurogenesis deficit in human neural stem cells
Source: PLoS Negl Trop Dis. 2021 Mar 3;15(3):e0009183. doi: 10.1371/journal.pntd.0009183 (PMC7959377; doi:10.1371/journal.pntd.0009183)

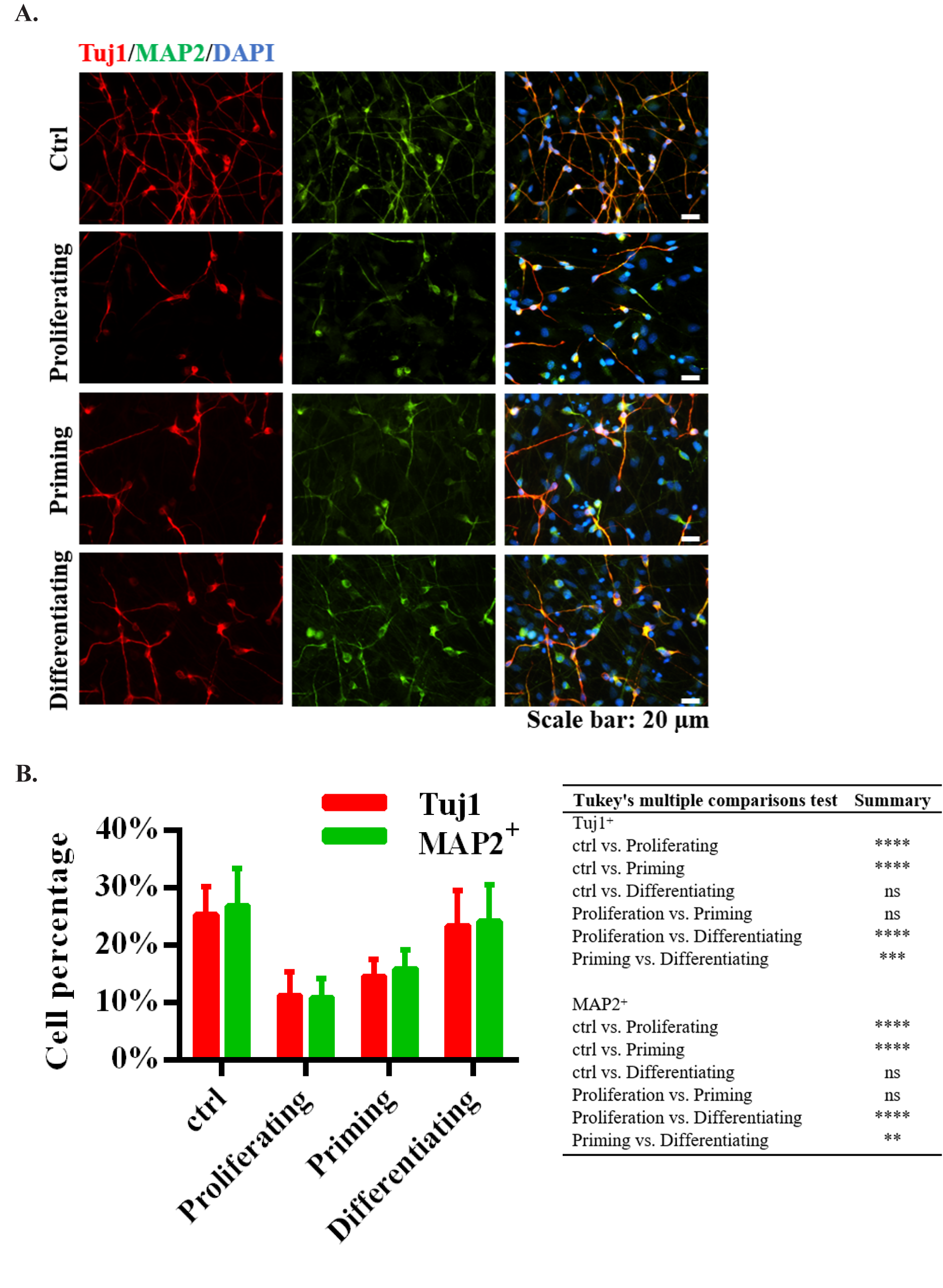

Supplement: S1 Fig — (A) Newly generated neurons were stained by Tuj1 (red) and MAP2 (green). Blue, nuclear counterstain. Scale bars: 20 μm. (B) Quantification data are presented as mean ± SD (n = 3), ** p<0.01, *** p<0.001, **** p<0.0001, two-way ANOVA with a Tukey’s multiple comparison test. (TIF) [file pntd.0009183.s001.tif]

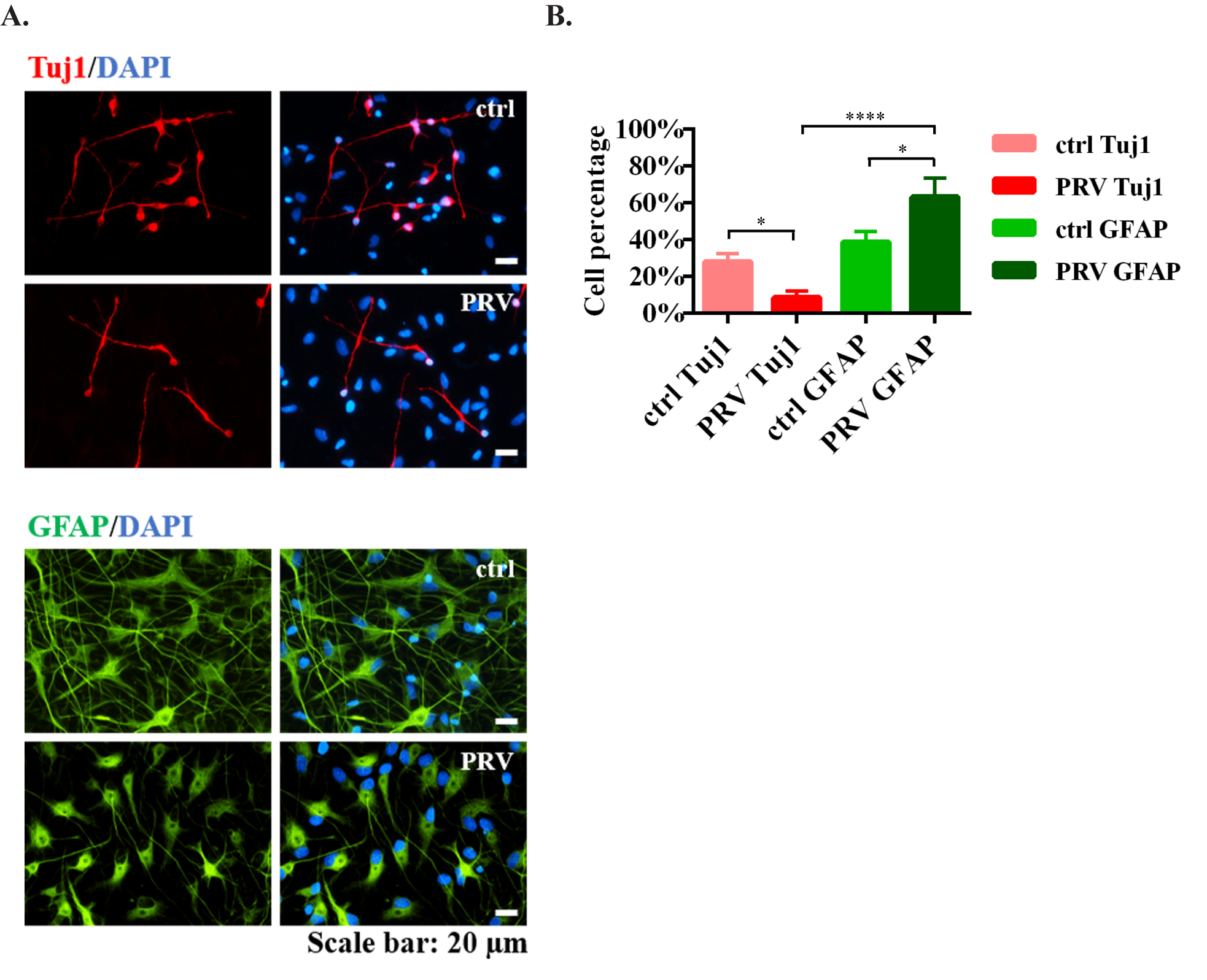

Supplement: S2 Fig — (A) Newly generated neurons were stained by Tuj1 (red), and astrocytes were stained by GFAP (green). Blue, nuclear counterstain. Scale bars: 20 μm. (B) Quantification data are presented as mean ± SD (n = 3), * p<0.05, **** p<0.0001, one-way ANOVA with a Dunnett’s multiple comparisons test. (TIF) [file pntd.0009183.s002.tif]

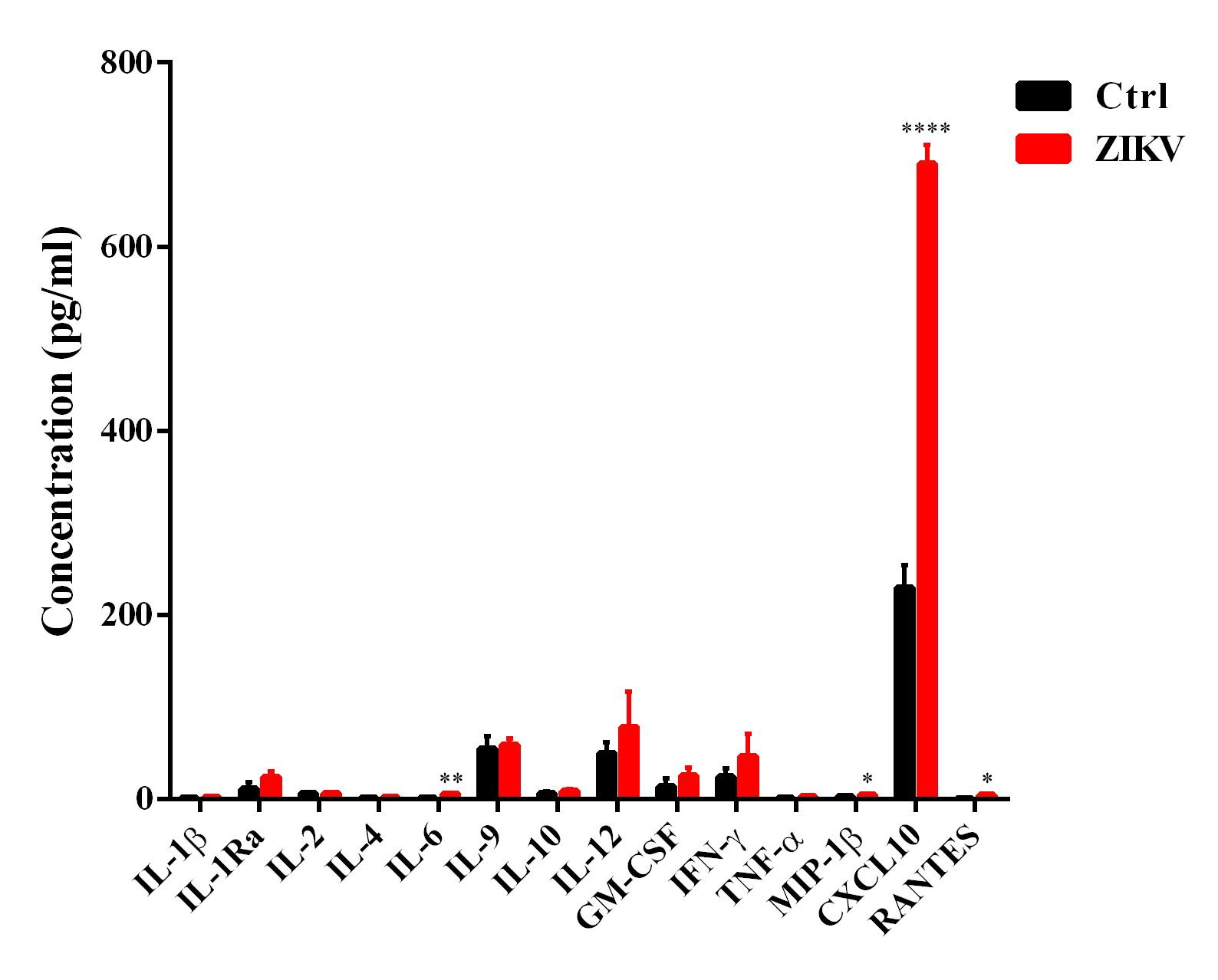

Supplement: S3 Fig — Culture medium were collected from ZIKV infected K048 cells. Concentrations of cytokines and chemokines in the medium were detected by Bio-Plex assay. Quantification data are presented as mean ± SD (n = 3), * p<0.05, ** p<0.01, **** p<0.0001, multiple t tests. (TIF) [file pntd.0009183.s003.tif]

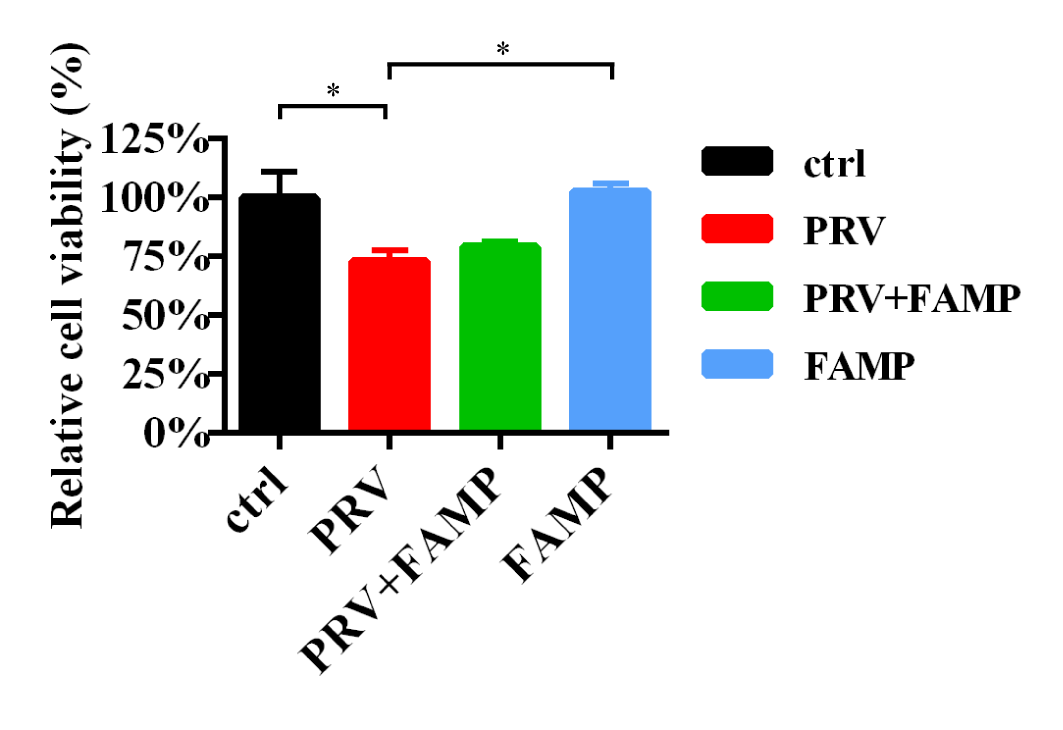

Supplement: S4 Fig — Cells were seeded in 96-well plates at a density of 5,000 per well. The viability was evaluated by the CellTiter-Glo Luminescent Cell Viability Assay kit (Promega) according to the manufacturer’s instructions. PRV, PRVABC59 strain of Zika virus; FAMP, Fludarabine. Data are presented as mean ± SD (n = 4), * p<0.05, two-way ANOVA with a Tukey’s multiple comparison test. (TIF) [file pntd.0009183.s004.tif]
