# Supplementary material for: Inhibition of innate immune response ameliorates Zika virus-induced neurogenesis deficit in human neural stem cells
Source: PLoS Negl Trop Dis. 2021 Mar 3;15(3):e0009183. doi: 10.1371/journal.pntd.0009183 (PMC7959377; doi:10.1371/journal.pntd.0009183)
Supplement: S1 Table — (DOCX) [file pntd.0009183.s005.docx]

**Supporting information**

**S1 Table.** **Primers used in this study**

| Primer | Sequence |
| --- | --- |
| GAPDH | F: 5′-AGGGCTGCTTTTAACTCTGGT-3′ |
|  | R: 5′-CCCCACTGATTTTGGAGGGA-3′ |
| IRF-7 | F: 5’-CAGATCCAGTCCCAACCAAG-3’ |
|  | R: 5’-GTCTCTACTGCCCACCCGTA-3’ |
| IRF-3 | F: 5’-TCTTCCAGCAGACCATCTCC-3’ |
|  | R: 5’-TGCCTCACGTAGCTCATCAC-3’ |
| B2M | F: 5′-CTATCCAGCGTACTCCAAAG-3′ |
|  | R: 5′-ACAAGTCTGAATGCTCCACT-3′ |
| TAP1 | 5′-GGGGACAGCTGCTGTTGGAT-3’ |
|  | R: 5′-AGTACACACGGTTTCCGGATCAAT-3’ |
| STAT1 | F: 5′-CTAGTGGAGTGGAAGCGGAG-3’ |
|  | R: 5′-CACCACAAACGAGCTCTGAA-3’ |
| STAT2 | F: 5'- CAGGTCACAGAGTTGCTACAGC-3' |
|  | R: 5'- CGGTGAACTTGCTGCCAGTCTT-3' |
| STAT3 | F: 5'- CATATGCGGCCAGCAAAGAA-3' |
|  | R: 5'- ATACCTGCTCTGAAGAAACT-3' |
| STAT4 | F: 5'- CAC CTG CCA CAT TGA GTC AAC TA-3' |
|  | R: 5'- TAA GAC CAC GAC CAA CGT ACG A-3' |
| STAT5a | F: 5'- GTTGGTCCTCTTGCCTCCTG-3' |
|  | R: 5'-GTCAAACCAGATCAGCTTTTACCC-3' |
| STAT5b | F: 5'- TGCTCTGCCCTGTTCAAGAAC-3' |
|  | R: 5'- CGCACTTCTCCTCTGTGACAG-3' |
| STAT6 | F: 5'- GTCTGGTCTCCAAGATGCCC-3' |
|  | R: 5'- ATATGCTCTCAAGGGTGCTGA-3' |
| DLX5 | F: 5′-ACCAACCAGCCAGAGAAAGA-3’ |
|  | R: 5′-GCAAGGCGAGGTACTGAGTC-3’ |

| Primer | Sequence |
| --- | --- |
| IRF-1 | F: 5′-TTTGTATCGGCCTGTGTGAATG-3’ |
|  | R: 5′-AAGCATGGCTGGGACATCA-3’ |
| TLR3 | F: 5’- CCTGGTTTGTTAATTGGATTAACGA-3’ |
|  | R: 5’- TGAGGTGGAGTGTTGCAAAGG-3’ |
| IRF-3 | F: 5’-TCTTCCAGCAGACCATCTCC-3’ |
|  | R: 5’-TGCCTCACGTAGCTCATCAC-3’ |
| DCX | F: 5'- TTGCCCTGTCTAATTTTGCC-3' |
|  | R: 5'- AAAAGGGGCACTTGTGTTTG-3' |
