# Supplementary material for: Inhibition of innate immune response ameliorates Zika virus-induced neurogenesis deficit in human neural stem cells
Source: PLoS Negl Trop Dis. 2021 Mar 3;15(3):e0009183. doi: 10.1371/journal.pntd.0009183 (PMC7959377; doi:10.1371/journal.pntd.0009183)
Supplement: Attachment — Submitted filename: Response_letter.doc [file pntd.0009183.s006.doc]

December 22, 2020

Dr. Gregory Gromowski

Associated Editor

Dr. Sunit Singh

Deputy Editor

Dr. Serap Aksoy

Editor-in-Chief

PLOS Neglected Tropical Diseases

Dear Editors,

We are delighted to learn your favorable decision on our manuscript entitled “Inhibition of innate immune response ameliorates Zika virus-induced neurogenesis deficit in human neural stem cells”. The reviewers’ efforts and helpful comments are also greatly appreciated. Accordingly, we revised the manuscript. Our point-to-point responses are shown below.

**Reviewer #1**:

1. Blocking TNF alpha with antibody has been shown to have protective role in mouse model to block cell death and increase survival. Does FAMP block cell death in NS/PCs?

FAMP did not affect cell survival/death in our system (page 12, lines 265-267). This is supported by a new supplemental figure (S4 Fig) in the Supporting Information. Cell viability assay is also added to Methods (pages 4-5, lines 88-90).

2. “Among those 2% of ZIKV-infected cells, nearly 90% were GFAP+ astrocytes, which were 8 times more abundant than Tuj1+ neurons”. Does this mean that ZIKV infects astrocyte more efficiently than neuron or there are more astrocytes in the culture? Or, ZIKV infection leads to the differentiation of NS/PCs to astrocyte other than neuron?

Both mechanisms are possible. Further clarification is now added (pages 9-10, lines 198-202; page 18, lines 396-397).

3. In addition to the innate immune response factors tested in this study, several other pathways components have also been reported. The authors should discuss why this study focuses on STAT1 while ignored the others, for example, IL6 and TNF alpha etc.?

As a hub transcription factor in the TLR3/IFN/MHC-I pathways, STAT1 controls the expression of numerous downstream factors that critically influence NS/PCs proliferation and neurogenesis. In our study, the transcription level of STAT1 was robustly increased by more than 10-fold after ZIKV infection, while others only altered moderately or mildly (Fig 4B). Additional discussion is now added (pages 16-17, lines 360-363).

In terms cytokines, a new supplemental figure (S3 Fig) is now added to show significant increases of IL-6 and other factors released from ZIKV-infected hNS/PCs in Results (page11, lines 235-241), together with clarification in Discussion (page 18, lines 390-394).

**Reviewer #2:**

•	Authors do not measure neuronal death, either by apoptosis, autophagy, or any other cell mechanism, which seems to be relevant in ZIKV infection during the neurogenesis process, in vitro or in vivo (Cugola et al., nature, 2016).

Cell viability data are now added in Methods (pages 4-5, lines 88-90) and Results (page 12, lines 265-266, S4 Fig).

•	Neurogenesis detrimental after ZIKV infection in neuro progenitor also pointed out other processes that should be discussed here (Rosa Fernandes et al., Frontiers in Cellular Neuroscience, 2019).

Agree with the reviewer, ZIKV infection alters the neurogenesis fate of NS/PCs, including both neurogenesis deficits and synaptogenesis impairments. Discussion is now added (page 18, lines 390-394).

•	NSC differentiation protocol used here differentiates both neurons and astrocytes?

Yes, our hNSCs were differentiated into both neurons and astrocytes, as shown in Fig. 1 and a new S2 Fig. Further clarification is now added in Results (pages 9-10, lines 198-202).

•	How many pics were used to quantify fig 2C? How many biological replicates were done?

These information are now added to Statistical Analyses of the Methods (page 7, lines 143-145).

•	Figure 3: What is the percentage of neurons and astrocytes in the differentiation protocol in control and ZIKV infected?

The percentages of neurons and astrocytes in control and infected cells are now added in S2 Fig, and clarified in pages 9-10, lines 198-202.

•	Fig 4F is written "poliferating"

Typos are corrected.

•	Authors suggested that the severity of neurogenesis impairment in hNS/PCs is caused by the extent of innate immune activation in the host cells. It would be essential to measure some cytokines and chemokines releasing in the cell culture system, not only by RT-qPCR. So, to associate the neurogenesis impairment of ZIKV infection only focusing on innate immune response, mainly based on FAMP inhibition of STAT1, is very interesting but sounds incomplete. The neurogenesis impairment is just based on the neuronal (TUJ1+ cells) counting?

A new S3 Fig is added to show ZIKV-mediated changes of cytokines and chemokines from infected hNS/PCs, which were measured in the cell culture medium by Bio-Plex assay (page 11, lines 235-241).

MAP2 is another neuronal marker indicating mature neurons (page 9, lines 190-191). Data are shown in a new S1 Fig.

Sincerely yours,

Ping Wu, M.D., Ph.D.

John S. Dunn Distinguished Chair In Neurological Recovery

Professor, Department of Neuroscience & Cell Biology
